# Review of Deep Learning Performance in Wireless Capsule Endoscopy Images for GI Disease Classification

**DOI:** 10.12688/f1000research.145950.2

**Published:** 2024-09-23

**Authors:** Tsedeke Temesgen Habe, Keijo Haataja, Pekka Toivanen

**Affiliations:** 1 School of Computing, Faculty of Science, Forestry and Technology, University of Eastern Finland, Joensuu, North Karelia, 70211, Finland

**Keywords:** Wireless capsule endoscopy, Deep learning, Transfer learning, Attention mechanisms, Multi-modal learning, Automated lesion detection, Interpretability and explainability, Data augmentation, Edge computing.

## Abstract

Wireless capsule endoscopy is a non-invasive medical imaging modality used for diagnosing and monitoring digestive tract diseases. However, the analysis of images obtained from wireless capsule endoscopy is a challenging task, as the images are of low resolution and often contain a large number of artifacts. In recent years, deep learning has shown great promise in the analysis of medical images, including wireless capsule endoscopy images. This paper provides a review of the current trends and future directions in deep learning for wireless capsule endoscopy. We focus on the recent advances in transfer learning, attention mechanisms, multi-modal learning, automated lesion detection, interpretability and explainability, data augmentation, and edge computing. We also highlight the challenges and limitations of current deep learning methods and discuss the potential future directions for the field. Our review provides insights into the ongoing research and development efforts in the field of deep learning for wireless capsule endoscopy, and can serve as a reference for researchers, clinicians, and engineers working in this area inspection process.

**Abbreviations**
CNN: Convolutional Neural Network
DICR-CNN: Domain-Invariant CNN
FPS: Frames Per Second
GCNN: Graph Convolutional Neural Network
GI: Gastrointestinal
KNN: K-Nearest Neighbors
LSTM: Long short-term memory
RNN: Recurrent Neural Network
VGG 19: Visual Geometry Group 19 layers
WCE: Wireless capsule endoscopy
YOLO-v4: You Only Look Once version 4

## Introduction

Wireless capsule endoscopy (WCE) is a minimally invasive diagnostic imaging modality that is used to examine the digestive tract. The procedure involves swallowing a small capsule equipped with a camera, which takes images of the digestive tract as it travels through the body. The images obtained from WCE are an important source of information for diagnosing and monitoring digestive tract diseases. However, the images are of low resolution and often contain a large number of artifacts, making their analysis a challenging task.

Artificial intelligence has shown remarkable diagnostic abilities in a variety of gastrointestinal medical image sectors ^1^ and health care sector. ^2^ Wireless capsule endoscopy is regarded as a valuable tool for diagnosing intestinal illnesses. Due to the millions of training constraints, existing DL (Deep Learning) approaches for pathology detection in WCE (Wireless Capsule Endoscopy) image are complex and computationally intensive. ^3^ Color and texture have a significant role in prominence target feature that aid in the recognition of abnormalities. ^3^ One of the WCE’s primary limitations has been that it captures many snapshots that should be sent to the attached screen and evaluated by physician: this takes a lot of time. Another drawback is the unclear line between lesions and normal tissues. ^4^

Deep learning techniques offer a lot of promise for helping doctors detect, find, and diagnose gastrointestinal disease with wireless capsule endoscopy. Several researchers have created image processing ^5^^–^^7^ and deep learning methods for finding and diagnosing disease from gastrointestinal tract problems using wireless capsule endoscopes over the last ten years. The main problem with capsule endoscopy is that images are obtained with inadequate lighting and the capsule camera moves around throughout the digestive tract, resulting in inadequate quality frames. ^1^

The most popular application of existing machine learning improvements in healthcare is Computer Aided Detection (CAD), ^8^^,^^9^ which is used to detect lesions, such as those present in the gastrointestinal tract.

Deep learning approaches, Supervised learning, and Transfer Learning methods were investigated in this study to better understand the situation and which types of CNN blocks and features can improve the classification and detection of GI pathology. To arrive at this conclusion, we first reviewed various recent papers in Table 1, held a discussion, and then compared accuracy in Tables 2 and 3.

**Table 1.** Aims, Deep learning features, datasets, result, and the recommended future works.

Objectives/Application	AI Features	Datasets	Result	Future work	Ref
For lengthy wireless capsule endoscopy (WCE) video, a new end-to-end temporal abnormality localization method was developed.	Graph Convolutional Neural Network (used GraphSage model) and for the image feature quality VGG 19 architectures & LSTM used as aggregated function	9 long videos	The overall result in nine length video the GCNN architecture performed accuracy was 85.9%. Sensitivity was 91.1%, specificity was 89.9%, and F-score was 90.5%	In the future, they should extend to a comprehensive graph organization using a multi label, multi-instance learning architecture, removing the requirement for temporal segmentation processing in each phase. The researcher did well at first, but as the future progresses, they will be able to boost their performance even more. There should be more disease categories.	^1^
Being investigated for use in building computer-aided diagnosis systems that detect abnormalities by binary classification of WCE images.	TICT-CNN is a CNN-based framework (Data augmentation	7259 normal and 1683 abnormal images	Selecting the proper color space can limit the number of trainable variables in half and reduce diagnosis time to just 0.02 seconds	Multiclass classification with large datasets to improve the classification of the pathology.	^3^
In wireless capsule endoscopy, gastrointestinal lesions and normal tissues are classified.	Branch3 effectively fused attention guided CNN (DenseNet121 trained on ImageNet)	Normal 10000 images, polyp 1000, ulcer 1000. (1800 train sets, 600 validation sets& 600 test sets)	Overall accuracy 96.50%, Accuracy, sensitivity, specificity (Normal: (98.17%, 97.00%, 98.75%), Polyp: (97.50% 95.50% 98.50%)) & Ulcer: (97.33% 97.00% 97.50%)	This article does not have a researcher future plan. However, I would recommend that the author focus on weakly supervised learning methods to increase performance in deep learning approaches.	^4^
The performance of a CNN approaches to sense lesions pathology using WCE and categorizing lesions of varying sternness was investigated in this study.	CNN: ResNet-50 pre-trained on ImageNet. The study applied image processing with a texture enhancement to increase the performance	A total of 7,744 images (Small bowel 4,972, colon 2,772)	The overall accuracy was 98.4%, the sensitivity was 95.7%, the specificity was 99.8%, The diagnostic accuracy: 98.5% for the small bowel and 98.1% for the colon	The AI elements described above were insufficient to figure out performance improvement. Deep learning, by its very nature, needs either enormous datasets or the most robust transfer learning techniques. As a result, individuals who are interested in improving detection must investigate deeper learning models.	^5^
Detect many lesions from WCE frames	Circular mask, ROI (regions of interest) (joint normal distribution model) by considering a threshold, a given probability density function (PDF), features (color, texture, and shape) from f RGB, HSV, and LAB color modes and the extracted: minimum, mean, variance, maximum, mode, median, entropy, and contrast, SVM (Support Vector Machine)	Kvasir-Capsule dataset	The overall accuracy was 95.4%, Recall was 95.2%, FNR was 4.8%, FPR was 4.3% and test time was 0.071	The researcher did not describe any future work. However, modern machine learning technologies should be used to carry out the work. The researchers use typical ways to carry out this study. For WCE, traditional procedures are not suggested.	^6^
To figure out SB (small bowl) cleansing values and test the algorithm’s effectiveness.	ROI segment by considering a threshold, features extractions (color, texture, and shape), classifier (SVM)	Total 400000 frames, 280000 for training and 120000 for testing	The overall accuracy was 93%, misclassification rate, 24.7%, Cohen’s kappa value, 0.672	The future work is not discussed by the researchers. However, I recommend that the researcher compare the performance of modern and traditional machine learning algorithms.	^7^
Prove that celiac pathology may be diagnosed using CAD without the usage of extremely sophisticated algorithms	Sobel filter, Cropping, convert to black and white image, contrast adjustment, binarization then classified by weighted KNN = 5	109 films (of 100 frames) are healthy and 65 come from celiac disease. 51 videos used for training, 51 videos for training and 7 videos for real time testing	Accuracy: 94.1% and F1 score: 94%	Because of the data set’s limitations, it is straightforward to suggest that it must be replicated by a larger data size to ensure that the investigations are valid.	^8^
Deep neural networks-based CAD tools are effective at detecting lesions in endoscopy,	Recurrent attention neural network developed (ResNet and VGGNet)	3498 images (2124 non-pathological, 1360 pathological and 14 inconclusive)	Sensitivity of 93%, a precision of 93.7%, and specificity of 95%	CNN is the most popular architecture above RNN. Because RNN focuses on text and video, while CNN focuses on enormous images. For CE images or video, I recommended that this author concentrate on CNN.	^9^
Automatic identification and Differentiation significant Colonic mucosal lesion	CNN models (Xception model trained on imageNet)	Total:9005 Normal: 3075, Blood: 3115, Lesion: 2815	Mucosal lesion: (Sensitivity 96.3%, Specificity 98.2% & Accuracy 99.2) Blood: (Sensitivity: 97.2%, Specificity: 99.9% & Accuracy: 99.6%), CNN trained (65 frames per second)	The best model was chosen for the work, but the researcher focused on the Xception model. It is preferable to include Transfer Learning methods in this model to improve model performance.	^10^
Classify gastrointestinal bleeding and non-bleeding classes	CNN models (used six convolutional layers alternated with max-pooling layers)	600 bleeding and 600 non-bleeding frames are used	Accuracy: 98.5%, Precision: 98.5% Recall: 98.5% AUC 0.9949, F1-score: 98.5%	Use bigger clinical image dataset with more lesions including vision tasks	^11^
Segmentation of bleeding, detection, and classification of GI tract diseases, and note of abnormality location	The researchers did not describe any models	Used KID database. Specifically, not described	The precision: 67.56%, recall: 73.03%, accuracy rate: 85.81%, and DICE (Diverse Counterfactual Explanations) rate: 69.99%, Jaccard Index (JI): 53.75%	Effective network architecture, more dataset, initial variable	^12^
The goal was to create an AI algorithm for detecting angioectasia Device-assisted enteroscopy photos automatically.	CNN: To improve performance, it needs to be redesigned with a decent future. CNN futures are not clearly described in this work	6740 images split into two:5345 normal mucosa, 1395 angioectasia	Sensitivity: 88.5%, specificity: 97.1% and an AUC: 98.8%	This paper does not go over CNN’s futures. So, how will this CNN architecture increase detection performance? We should examine various recent AI futures with CNN to evaluate its performance.	^13^
Study on Transfer Learning features for image analysis.	Transfer learning: (AlexNet, ResNet, VGGNet, and GoogleNet)	No used	Most researchers used ImageNet for medical image categorization. The researcher did not reach a conclusion due to most unwell paper found for review	uses Transfer learning features from other domains like not ImageNet dataset to classify and detect images.	^14^
In the WCE images, find the lesion regions and improve the classification accuracy.	CNN: ResNet-50 (self-attention) and convolutional stem mechanism	Kvasir-Capsule dataset	Overall accuracy 95.1%	When we look at the accuracy values, the article’s result is good, however the future should be detected and diagnostic by deep learning. The article will go on to discuss how to train and assess tiny datasets with deep learning and transfer learning.	^16^
To obtain a better model for domain-specific tasks using wireless capsule images.	Self-Supervised learning: a ResNet-50 and dense layer	Used 49 WCE videos that are not labeled	AUC 95.00%, final accuracy was 92.77%	Researchers want to focus on various SSL architectures in the future to see whether they can improve the performance of downstream operations.	^17^
A new automatic classification approach for Gastrointestinal problems that is easy to understand.	CNN: XAI makes the decision, and Bayesian optimization and Darknet53 and Inception-V3 architectures are used as AI features to boost performance.	Benchmark dataset Kvasir is used for test performance, but dataset scale or number is not known	The overall accuracy of Bayesian optimized SVM on CNN and XAI is 97.0%: recall 93.9, precision 93.9, F1-Score 93.9, AUC 0.997	The complexities of the model must be considered in future research, and imbalanced datasets make the training task more complicated. The aim for future researchers will be to overcome this constraint.	^18^
To improve binary classification of WCE images	To test the efficacy of CNN, a pre-trained Inception network feature was used. (DICR-CNN: dilated input context retention CNN)	7062 images for train, 940 images for test and 940 images for validation	Overall accuracy 0.96, sensitivity 0.93 and specificity 0.97	For the algorithms used, the data set is quite small. It would be preferable to expand this study to include Transfer learning approaches and the use of different CNN features to improve the performance of multi-pathology detection and classification in WCE images.	^19^
This research proposes an AI technique for accurately classifying Gastrointestinal tract datasets with a limited size of annotated data.	LSTM-CNN with AlexNet, Google Net, and ResNet architecture. The LSTM block is used in the CNN classifier.	A total of 2000, 4000, and 6000 images have been tested individually	Using 6000 images, metric Accuracy: 98.05% sensitivity: 98.05% specificity: 99.72%, precision: 98.05%, F1-Score: 98.05% Matthews Correlation Coefficient: 97.77%	Future research must investigate CNN-specific Long-short term memory layers for all highly improved detection in CNN theories and techniques.	^20^
This paper describes pathology classification techniques for a GI tract classification problem with minimal labeled-data and several imbalanced classes.	GoogLeNet, AlexNet, and ResNet are architectures for improved CNNs (Convolutional Neural Networks) with LSTM blocks.	In the study, datasets 2500, 5000, and 7500 were evaluated separately	97.90% is overall accuracy. The maximum values for largest scale of datasets with Sensitivity 92.32%, Specificity 99.10%, Precision 94.46%, F-score 92.64% are improved	No proposed future work, but if LSTM is used for capsule endoscopy images, I recommend that the author concentrate on video frame level localization. In this future study, they should employ a video dataset for categorization. Additionally, before training datasets to detect the effect of CNN, the job should incorporate the influence of picture enhancement.	^21^
Binary classification of wireless capsule endoscopy (lesion and normal classes)	CNN (Inception- Resnet-V2) by using transfer learning futures)	200000 images	The overall accuracy was 98.1%, the sensitivity was 97.7%, the specificity was 98.5%, probability score cut-off of 0.541	We have seen some excellent work in this article. The researchers used AI elements such as transfer learning methodologies to try to increase CNN performance. However, this performance will require an increase in the categorization number.	^22^
For improved detection, various image processing (traditional) methods are combined with a data augmentation approach to improve modern methods.	CNN (use the VGG16, ResNet-18, and GoogLeNet models that have been pre-trained) and fully connected and output layer.	6702 images for 8 classes	Accuracy: 96.33%, recall: 96.37%, precision: 96.5%, and F1-measure: 96.5%, the VGG16 model came out on top	Using a larger dataset that has been labeled by a larger group of specialists is one way to improve pathology detection.	^23^
To improve multi-class of various disease classification in capsule endoscopic image of the gastrointestinal tract in the situation of imbalance datasets.	Abnormal feature attention relationship network by using feature addition, concatenation, and bilinear merge.	2000 KID data set	The 2000 tagged capsule endoscopy images from the KID database with diverse illness groups yielded an overall classification accuracy was 98.78%	Although the researchers did not intend to include this element in their analysis, I recommend that they concentrate on learning to perfect and compare. Furthermore, standing examine how to learn new pathology from a few samples is a wonderful idea for a Few-shot learning approaches.	^24^
As the underlying architecture, recognize tiny polyp border in WCE images using the precision-vs-speed trade-off.	Multiscale pyramidal-FSSD (CNN features VGG16 for backbone network with SSD (Single Shot Detector) layer.)	120 polyps’ images and 181 normal images	In VGG16 network the testing speed (FPS) is 62.75 mAP and Accuracy is 93.4%	Multiscale pyramidal -FSSD will be upgraded in the next plan to enhance performance on datasets, or even both, adopting more powerful backbone networks such as DenseNet121.	^25^
From capsule endoscopy imaging, many small intestinal lesions with distinct haemorrhagic potential were found and differentiated.	CNN (Xception feature on ImageNet)	A total of 53 555 capsule endoscopy images	Accuracy: 99%, sensitivity: 88%, specificity: 99%, PPV: 87%, and NPV: 99%	To be sure, the CNN model architecture must be state-of-the-art. For their outcomes, the writer does not compare the performance to the existing CNN performance.	^26^
Create a CNN model for finding and classifying vascular lesions in WCE pictures with varying hemorrhaging probabilities.	CNN (Xception)	Normal: 9525 red spots:1026, varices:1037. Total: 11588	Accurate: 94.4%, sensitive:91.8% and specificity:95.9% for vascular lesions Sensitivity: 97.1% and specificity: 95.3%for detection of red spots. Sensitivity: 94.1% and specificity: 95.1% for varices detection	In the future, researchers should investigate more high-quality datasets and classification numbers to see if this model can be used to extend transfer learning approaches.	^27^
The goal of the investigation is to investigate the performance of CNN algorithms for multi-label pathology detection.	The spatial features are obtained using ResNet50, and the temporal features are obtained using residual LSTM blocks.	14 colorectal disease and artifact from 455 video by 28,304 frames	Precision: 61.6, recall: 54.6%, F1-score: 55.1%, specificity: 95.1%	To improve performance on frame-level localization, the researcher should enlarge the dataset and collect more movies in the future, as well as broaden the examination into pathologist domain knowledge.	^28^
Created an AI approach based on a CNNs (Convolutional Neural Networks) features for detection of blooding or hematic residues in CE images.	CNN developed with the Xception.	Total image 5825 (luminal bleeding or hematic: 2975 normal case: 2850	Accuracy: 96.6%. Sensitivity: 99.8%, specificity:93.2%, and Positive and negative predictive: 99.8%	The researcher should explore the model of CCE and its limitations in the current features because the CCE limitations were not clearly explained. Furthermore, more CNN aspects must be addressed or compared to figure out CNN’s outcome or performance. When using a small dataset with CNN, the transfer learning possibility is ideal.	^29^
to create a deep learning approach that uses CNNs (Convolutional Neural Networks) to detect hookworms in WCE images automatically.	CNN with on a You Look Only Once-Version4 (YOLO-v4)	Hookworms’ images: 531, small-bowel images: 10,529	The overall accuracy was 91.2%, the sensitivity was 92.2%, the specificity was 91.1%, probability score cut-off of 0.485	This research proves superior performance in detecting a single disease. However, multi-pathology detection in WCE needs to be expanded. Multi-class datasets with big scale datasets should be the focus of future research.	^30^

**Table 2.** The accuracy of CNN Methods based on datasets and AI characteristics is compared.

Architecture name	Domain Method	Dataset	Classes	Class name	Transfer learning	Accuracy %	Sensitivity %	Specificity %	Ref
Xception	CNN	3115 images	2	Blood	No	-	97.2	99.2	^10^
		2815 images		Mucosal Lesions	No	-	92.0	98.5	
		53555 images	Multiple	Small bowel lesions	No	99.0	88.0	99.0	^26^
		9525 images	3	vascular lesions	No	94.4	91.8	95.9	^27^
		1026 images		Red spots	No	-	97.1	95.3	
		1037 images		Varices	No	-	94.1	95.1	
		5825 images	2	Luminal blood and Normal	No	96.6	99.8	93.2	^29^
VGG16	CNN	6702 images	8	8 pathology types	Yes	96.33	96.37		^23^
		201 images	2	Polyp and Normal	Yes	93.4	-	-	^25^
Inception- Resnet-V2 model	CNN	400000 images	Multiple	Lesion and normal classes Detection	Yes	98.1	97.7	98.5	^22^
ResNet50	CNN +LSTM	445 videos	14	14 colorectal disease	Yes		54.6	95.1	^28^
ResNet	CNN	2500 images	Multiple	GI track	yes	93.02	85.83	98.34	^21^
		5000 images				95.45	88.26	98.71	
		7500 images				97.90	92.32	99.10	
	RNN	3498 images	2	Pathological and non-pathological	No	93.7	93	95	^9^
	CNN+LSTM blocks	2000 images	8	Z-line, pylorus, cecum, esophagitis, polyps, ulcerative colitis, dyed and lifted polyps, and dyed resection margins	Yes	93.01	93	99	^20^
		4000 images				95.43	95.43	99.35	
		6000 images				98.05	98	99.72	
AlexNet	CNN+LSTM blocks	2000 images	8	Z-line, pylorus, cecum, esophagitis, polyps, ulcerative colitis, dyed and lifted polyps, and dyed resection margins	Yes	91.35	91.35	98.76	^20^
		4000 images				94.37	94.07	99.15	
		6000 images				97.50	97.22	99.60	
		2500 images	Multiple	GI track	yes	90.37	83.16	97.97	^21^
		5000 images				94.50	87.05	98.61	
		7500 images				96.95	89.48	98.91	
GoogleNet	CNN+LSTM blocks	2000 images	8	Z-line, pylorus, cecum, esophagitis, polyps, ulcerative colitis, dyed and lifted polyps, and dyed resection margins	Yes	91.70	91.70	98.81	^20^
		4000 images				95.00	95.00	99.29	
		6000 images				96.80	96.80	99.54	
		2500 images	Multiple	GI track	yes	90.28	83.08	97.86	^21^
		5000 images				94.58	87.19	98.76	
		7500 images				97.15	90.51	98.85	
YOLO-v4	CNN	531 images	2	Normal and Hookworm	No	91.2	92.2	91.1	^30^
VGG 19	GCNN +LSTM	9 videos				85.9	91.1	89.9	^1^
DenseNet121	Branch3 effectively fused attention guided CNN	10000 images	3	Normal	No	98.17	97	98.75	^4^
		1000 images		polyp	No	97.50	95.5	98.50	
		1000 images		ulcer	No	97.33	97.00	97.50	
Inception	DICR-CNN	8942 images	Multiple	Binary classification in WCE images	No	96	93	97	^19^

**Table 3.** The following are the top five articles.

Methods	Accuracy %	Ref
Xception	99	^26^
DenseNet121	98.17	^4^
Inception-Resnet-v2	98.1	^22^
ResNet	98.05	^20^
AlexNet	97.50	

### Deep learning

The Wireless capsule Endoscopy Deep Learning implementation detected, red spots, vascular lesionsulcers, small bowel lesions, mucosal lesions, polyps, celiac disease, bleeding, and hookworm. In most existing papers, Deep Learning applications with various CNN features were used to detect all 14 colorectal diseases. Because Deep Learning in Artificial Intelligence plays a significant role and performs well in medical image identification and processing, it has gained impressive performance improvements in the identification of a variety of diseases in Wireless Capsule Endoscopy.

The CNN models with Xception and ImageNet features show reliable performance of transforming the color space, which completely transforms one image into a new one and then the CNN ^1^ can extract features for classification.

The texture, color, and shape qualities ^3^^,^^10^^,^^11^ were used in the training. Since color can influence training results, image processing (e.g., white balance adjustment) was needed to reduce color alterations. ^10^ HSV-S, Gray, and Lab-b components were employed as source data for training in the HSV, RGB, and Lab color models, respectively. ^10^ CNN in WCE images with YOLO-v4 ^11^ feature showed high-accuracy, fast, and error-free method for finding intestinal anomalies in WCE images and classification. The models constructed with CNN with Xception and ImageNet yield the extracted features and three classifications. The performance was evaluated by using sensitivity and specificity. In the future the researcher plans to improve the performance of the deep neural network by using effective networks construction (architecture), scale of datasets, initial variables, and NN algorithm generalization. ^12^

On-the-fly data augmentation and color space transformation are performed by TICT-CNN, accompanied with binary classification of the frames. ^3^ The use of Deep learning to wireless capsule endoscopy ^3^ and Device-assisted enteroscopy ^13^ could have a significant impact on how patients with gastrointestinal hemorrhage are treated.

AI features, including LeNet, AlexNet, ^14^^,^^15^ VGGnet, ^14^ GoogLeNet, ^14^ and ResNet, ^14^^,^^16^^,^^17^ DenseNet121, ^14^^,^^18^ Xception, ^14^^,^^18^^,^^19^ have been recently the most used in wireless capsule endoscopy image. The most widely improved convolutional CNN architecture is Alexnet. ^15^^,^^20^^,^^21^ In some application AlexNet has outperformed architectures in terms of improving CNN performance.

In the absence of pixel-level labels, TICT-CNN ^3^ is used when datasets-level in WCE image labels are used as weakly annotated-data for training. Self-supervised learning ^17^ is a practical method for dealing with the problem of insufficient training data and annotations. SSL performance is good in the ResNet-50 design with dense layer. ^17^

The performance of CNN in wireless capsule image pathology detection/classification is typically restricted because of the small, labeled CE (capsule endoscopy) image datasets. Transfer learning (TL) ^22^^,^^23^ is beneficial for several tough pathology identification operations, and it is one solution to machine-vision problems when only restricted CE images are typically available due to patient medical imaging data privacy concerns. We should research the best ways to learn and verify pathology detection with a few datasets to solve such problems.

Transfer learning approaches with a pre-trained model were applied with ImageNet datasets, and the rectified linear unit (ReLU), ^14^^,^^17^^,^^24^ dense layer ^17^ was used as a transfer function for transfer learning. The VGG16 model really does have the highest Matthews Correlation result of 95% and Cohen’s Kappa score of 96% when compared to other algorithms. ^23^ During the training datasets of the amplified Kvasir-version-2 datasets, fine-tune three key pre-trained CNN are VGG-16, ^25^ ResNet-18, ^23^ and GoogLeNet. ^23^ VGG-16 consists of 16 layers, 3 of which are 3 dense layers and 13 convolution layers. ^23^ In comparison to ResNet-18 and GoogleNet, VGG16 has performed better in this architecture in. ^23^ Using a larger dataset that has been labeled by a larger group of specialists is one way to improve pathology detection. ^23^ However, to overcome the challenges of obtaining a huge dataset, we need use the best transfer learning techniques available, such as the few shots learning approach, which can learn new things from current datasets.

Although the dataset in ^22^ is 30 times larger than in, ^23^ the author’s result in 6 is better than in 7 because with a few datasets, the researcher used a better architecture than in, ^23^ which solved the issues of gathering large medical datasets. However, a huge dataset ^23^ helped to improve the effectiveness of CNN features in wireless capsule endoscopy. Xception model with ImageNet ^10^^,^^13^^,^^26^^,^^27^ was able to reach higher levels of accuracy than ^23^ when we compare as well as great image processing results with superior performance. We hope that their findings will contribute to the wider adoption of AI (Artificial Intelligence) technology in the field of WCE for researcher who wants to work on Deep learning with transfer learning approaches. The researcher employed the EFAG-CNN ^4^ with three branches: branch 1, branch 2, and branch 3. Initially, AI DenseNet121 ^4^ built on ImageNet was trained using branch 1 and branch 2, then fine-tuning approach was employed to improve performance and convergence speed. Branch1 has been used to concentrate the lesion region, branch 2 has been used to extract useful features from the lesion area, and branch 3 has been used to combine global and local features to get the final prediction result. ^4^

When the available labeled datasets are insufficient to produce a supervised model with improved performance, weakly supervision ^1^^,^^28^ is seeking to gather more labeled data for supervised training and modeling. The labeled data that is accessible is noisy or comes from an unreliable source. Weakly supervise learning used by most researchers for the long video localization in wireless capsule endoscopy. GCNN (Graphical Convolutional Neural Network), ^1^ unlike CNN, is designed to work with non-Euclidean structured data. The author gathered extensive videos, which will be converted into nodes Count. GCNN ^1^ performed a node count in the form of non-Euclidean structured data (space). For the AI characteristics and architecture, most of the researchers employed CNN, which means they used Euclidean space. The challenge of poorly labeled data is addressed by weakly labeled datasets. ^28^ The film produced by Wireless Video Capsule Endoscopy (WCE) holds up to 52000 images. Labeling these datasets takes a long time, which is why doctors do not do it. They can make a remark about a certain frame (and that this label is for a time-region in the video). This writes down that the data is poorly labeled. There is also a substantial correlation between frames that can be exploited. Spatial and temporal features, as well as memory (LSTM (Long Short-Term Memory) and attention, used in. ^28^

GraphSAGE ^1^ is good at predicting the encoding of a new node without the need for re-training. This feature is used to manage CNN training by shortening the time it takes, but only for capsule endoscopy video, not images. The researcher begins, with temporary segmentation of the long video into consistent, similar. Then they represented as a graph, the frame in the capsule endoscopy video as the node by connected the relation by the graph’s edges. ^1^ To make this relationship we used a variety of identical measures such as correlation, cosine similarity, and Euclidean distance among the nodes of the graph. ^1^ In weakly supervise learning with GCNN used pretrained VGG19 ^1^ trained on huge ImageNet datasets after that finetuned to remove inadequate lighting and inferior quality in the video frames. On large graphs, GraphSAGE ^1^ offers interactive and dynamic learning. LSTM, Mean, and max pool, are examples of aggregation functions that can be used in GCNN. Long Sort term memory ^1^ and max-pooling for few-shot learning ^24^ outperformed competitors in.

### Datasets

The protection of patient information is one of the most difficult aspects of data acquisition. As a result, we are unable to gate huge datasets from medical fields. To deal with data scarcity, most researchers use the augmentations feature, and transfer learning techniques can also be used to reduce datasets or train CNN networks with minimal datasets. To generate extra new supplementary datasets with varied features of the images, augmentation approaches ^1^^,^^3^ are applied. Few-shot learning ^24^ is another method for improved classification by training a limited number of labeled datasets. As summarized in Table 2 some authors employed Transfer learning algorithms and functions to tackle dataset scarcity. ^10^^,^^20^^,^^21^^,^^29^ The largest datasets were 400,000 with Inception- Resnet-V2 architecture ^22^ and performed 98.1 accuracy, while the smallest datasets were 201 with VGG 16 network architecture ^25^ and performed 93.4 accuracy in this systematic investigation.

A Few-Shots Learning is a core solution to the delinquency of classifying wireless capsule endoscopy (WCE) images with few labeled data. ^24^ To increase the performance of the Abnormal Feature Attention Relation Network a few-shot learning applied in ^24^ with a few datasets. In wireless capsule endoscopy, feature addition, feature concatenation, and bilinear merging improve this learning architect for foreground abnormal feature enhancement methods. ^24^ When compared to the bilinear merging strategy, the abnormal feature attention module (AFA) ^24^ is more effective, improved by 10.74% across Relation Network (RN). Recognize small polyp boundary in WCE photos using the Multiscale pyramidal FSSD (Fusion Single Shot Detector) ^25^ approach as the underlying architecture, balancing precision, and speed.

## Methods

The ultramodern survey began with an analysis of relevant keywords based on subjects or topics. For searching similar and relevant publications from official databases, we found key terms. CNN, Deep learning, Transfer learning, and wireless capsule Endoscopy are just a few main key terms. We used a combination of key terms with “AND” and “OR” logical operations to find related articles. Using the University of Eastern Finland article searching database platform link https://primo.uef.fi/, we obtained 222 peer-reviewed publications from Association for Computing Machinery, PubMed and other international publishers based on the search strategies. The peer review papers were collected from the last 10 years of publication, which began in 2013 and ended in 2022. However, for our inquiry, we chose the most relevant and recent 30 open articles.

Most peer-reviewed papers were from MedPub publishers. Most of the publications investigated on CNNs (Convolutional Neural Networks), which are a type of DL technology that is widely used for WCE images. As a result, we chose the most recent and most relevant journal articles for the survey. We primarily focused on how to overcome the challenges of low light, shadow, low resolution, and noise in wireless capsule endoscopy for enhanced pathology identification using Deep learning algorithms.

AI features, we construct a summary of the methods, with outcomes based on Accuracy, Sensitivity, and Specificity. We compare the results of the top 18 articles development models based on dataset scale and transfer learning features in the next steps.

Finally, we compare the top five findings to decide which AI features and CNN architecture to propose for improved pathology in WCE image using Deep learning methods. Sensitivity and specificity had been evaluated as a group. We can look at CNN’s network design and summary in Table 2 for more information. This will aid in figuring out the best wireless capsule endoscopy feature and procedures.

## Discussion

Using wireless capsule endoscopy images, deep learning models have shown promising accuracy in the classification of gastrointestinal disorders. Research findings indicate that the average accuracy of these models is 99%, demonstrating their potential to support precise diagnosis. But managing nuanced illness symptoms and intricate anatomical variances presents difficulties, necessitating ongoing model reliability development. The pathology map estimator and the classification network are cooperatively tested and trained to enhance detection results by employing ResNet-50 (self-attention) and convolutional stem mechanism features. ^1^ Performance of the model is greatly influenced by the features of the dataset. A stronger generalization is facilitated by large, diverse datasets with well-annotated images. Performance might be impacted by problems including unequal disease representation and differences in imaging conditions, which emphasizes the need for well selected datasets. The lesion part or border is very thin in the wireless capsule endoscopy image, making it difficult to distinguish between the lesion and the normal part of the image in the WCE image. To illustrate the anomalous pattern, most of the researchers concentrated on the entire wireless capsule endoscopy photos. To distinguish this exceedingly small lesion boundary from the rest of the image, as the researcher said in, ^1^ you must use the best representation learning approaches by focusing on the lesion regions. The authors used the CNN features with a unique lesion attention map estimator model ^1^ and ResNet-50 (self-attention) ^1^^,^^28^ to solve this anomalous pattern. Some of the researchers also use balancing network depth, image resolution, model performance, and parameter complexity reduction to improve the binary classification of WCE images. ^19^ Some scientists presented a unique pathology sensitive deep learning methods for frame level variance identification and multi label categorization of diverse colon diseases in CE data. ^6^ Weekly supervised model ^1^^,^^28^ mostly used for multi-label disease identification. According to the work, attention-based deep MIL ^28^ was trained end-to-end on poorly labeled image using video labels rather than comprehensive frame by frame annotation, but the results are poor based on LSTM. ^28^ The proposed approaches include ResNet50-designed for spatial features and residual LSTM blocks for temporal features, ^28^ a learned temporal attention module for final label identification, and self-supervision ^1^^,^^28^ method to enlarge the distance between pathological classes, as well as a self-supervision method to maximize the remoteness between pathological classes. AlexNet possesses these features or architecture are good approaches for Endoscopy pictures because it is hard to obtain a significant quantity of datasets. ^21^ So, if we have a little dataset, we can increase CNN network performance by utilizing ResNet and AlexNet ^20^ and if we have a huge dataset, we should utilize xception and DensNet121. ^4^ However, DensNet121 in Ref. 4 also performs well with limited datasets outperformed architectures in terms of improving CNN performance about image processing problems. Addition of CNN layers, number of datasets, number of Epoch, image size, color channel (reduction), transfer learning using pre-trained models such as YOLO and ResNet ^14^^,^^30^ are some of the elements affecting the performance of deep learning progress that must be examined in the research. Table 2 shows how the Xception ^26^ design helps CNN achieve high accuracy (99%) with datasets 53555. With DenseNet121 ^4^ network design and 1000 datasets, the highest accuracy is 98.17%. Transfer learning techniques were used by both researchers. ^22^^,^^25^ However, according to the journal study, 93.4% accuracy was achieved due to the researcher’s employment of data-efficient learning methods with tiny datasets. Comparative studies show that deep learning outperforms classical techniques in identifying complex patterns in capsule endoscopy images. Even if deep learning is superior for feature learning, conventional techniques are still useful in situations when there is less data. Comprehending the advantages and drawbacks of every methodology is vital for making knowledgeable therapeutic judgments.

To advance the research, disciplinary collaboration is crucial. Creating knowledge-sharing platforms, publicly available datasets, and uniform assessment metrics encourages collaboration to advance deep learning in wireless capsule endoscopy. Collaboration among researchers, clinicians, and tech developers fosters a more comprehensive understanding of the problems at hand and speeds up progress.

### Image quality in WCE

It is not always beneficial to enhance the WCE images to a higher resolution but the contrast and the texture can make a huge difference especially for the model when trying to detect small lesions. As pointed out by other researchers, low contrast in WCE images will result in important features being washed out and thus making it extremely hard for deep learning models to differentiate between healthy and diseased tissues. For example, ulcers and polyps are characterised as small elevated or flat areas that are morphologically slightly different from surrounding tissue; they may be extremely difficult to identify in low contrast images where tiny differences in the texture compared to surrounds are suppressed. Models like the CNNs can also be able to rectify the texture and contrast in order to make these distinguishable differences make this classification possible and accurate. Some of the enhancements like the histogram equalization, and contrast-limited adaptive histogram equalization (CLAHE) can enhance the sensitivity of the model and minimize false negative results of lesion detection.

### Challenges and limitations

Deep learning has exhibited impressive results in segmentation and lesion detection for WCE, but there are several constraints towards the classification problem. For instance, the current models in classification of GI diseases for instances Crohn’s disease and ulcers are difficult to be achieved because of the high variation of lesion appearance as well as appearance of artifacts. One of the critical issues is inclusion of the different types of lesion with similar appearance (e.g., inflamed tissues resemble normal folds) mainly due to low resolution and contrast in WCE videos. Further, due to the imbalance of the datasets wherein some of the GI diseases are less predominant, model prediction tends to give preference to the widespread disease, such as polyps. Bubbles and thin food residues present on tissue surfaces, and motion blur also complicate the models’ task and misclassify these artifacts as pathological. As such, the future work needs to direct its efforts towards the creation of stronger classification models that would encompass the specifics of the given domain as well as employ improved pre-processing mechanisms that would eliminate the interference of artifacts.

### Future directions

For the extension of deep learning for WCE, future studies should also center on semi-supervised and data-efficient learning methods which can effectively work under the constraint of scarce labeled data. For example, the use of other approaches such as self-training or consistency regularization as part of the semi-supervised learning (like soft-teacher model ^31^) can utilize large datasets of unlabeled WCE images and hence minimize on the use of laborious procedures of annotation. Moreover, there are some promising directions which will potentially improve the models in the low-data conditions – transfer learning, where pre-trained models on large and diverse medical databases are retrained exactly for the WCE applications. A future work is feasible to apply the TCNs or RNNs for the real-time analysis of WCE data because these networks are suitable for video anomaly detection. Another area of focus for researchers should also be the establishment of comprehensive WCE datasets and reference standards that will enable easier comparisons among these models and help scientists in the various AI fields, clinicians, and engineers to collaborate effectively.

Due to challenges in diagnosing gastrointestinal diseases using WCE, there is a need to have collaborations between artificial intelligence, gastroenterologists, and medical imaging specialists. The building of clinically relevant AI models for WCE presupposes the expertise in the method of deep learning as well as the problem-oriented approach toward clinical decision making. There are several strategies which can be employed to encourage interdisciplinary collaborations; these include the use of data shared across disciplines, cross-disciplinary workshops and cross-disciplinary research grants. For instance, clinicians can help AI researchers interpret certain disease manifestation patterns for the specific GI diseases, which, in turn, allows researchers to tweak their algorithms to enhance the models’ performance. Also, engineers can help made hardware and software solutions used in AI as efficient as possible, and to adapt them to be implemented in the clinic.

## Result

Table 3 summarizes the final five best-performing articles based on the accuracy and techniques described in Table 2.

According to the researchers, two selected strategies in the above table show the best results. However, the approaches chosen take advantage of the largest database, 53555 ^25^ and 1000 images for DensNet121, ^4^ as well as data-efficient learning methods. 400,000 images with an Inception-ResNet-v2 feature score of 98.1% are the largest datasets in this state of the art. This method requires more datasets to achieve 99 to 100% accuracy, since 400,000 photos can get 98.1 when compared to the first two articles, which needs many datasets and a revision of the current method. With 6000 photos, ResNet and AlexNet ^20^ supply 98.05 percent and 97.50% accuracy, respectively. Finally, utilizing AlexNet and ResNet, ^22^ the researcher achieves high accuracy with a limited dataset. Table 3 (shown below) has more information about the results.

There are challenges in integrating deep learning models with current workflows, ethical concerns, and regulatory compliance when moving these models from research to clinical application. In order to develop guidelines for safe and efficient deployment, researchers, healthcare providers, and regulatory bodies must work together to address these challenges. In order to solve present issues, future developments might make use of cutting-edge technology like federated learning and explainable AI.

Working toward a future in which deep learning models are useful instruments for decision support, frameworks for cooperation between AI and medical professionals are taking shape. Efficient decision-making and improved diagnostic precision can result from combining the analytical power of AI with the clinical expertise of medicine.

In the present comparison of different deep learning methods (Refer Tables 1 and 3) for WCE, factors such as accuracy and specificity should be complemented with a focus on work flow implications. For instance, YOLO-v4 which is the latest version of You Only Look Once model is excellent for real-time object detection, fast and accurate; it can be used to detect anomalies while WCE videos in real-time and give feedback to clinicians performing the procedures. However, the drawback of such a quick approach is the reduced ability to detect smaller or less conspicuous lesions. On the other hand, the models that are based on the transfer learning like the models pre-trained on ImageNet may have better accuracy in the classification of the lesions but this poses the need for many computational resources in terms of time and money thus making it very hard or rather not very practical in most of the clinical settings. Nonetheless, the issue of interpretability is still an open problem as most are deep learning models are ‘black box’ in nature and hence needs to be enhanced using attention mechanisms and explainable AI so that clinicians can rely on the decisions made by the such models to arrive at more informed and accurate clinical decisions.

## Conclusion

Wireless Capsule Endoscopy is a medical imaging technique used to have a view of the GI tract causing malfunction and few researches have been documented to have applied deep learning techniques to analyze WCE images in recent years for different applications for instance segmentation and registration.

Nonetheless, according to our recent research described in this paper, there is still much room for enhancement especially in the identification and differentiation of pathologies in the GI tract.

One potential area for improvement found is in learning from data efficiently and the use of better forms of artificial intelligence. Since the medical data annotation is expensive and time-consuming it is essential to improve the efficiency of the learning methods. Computer aided diagnosis, few sample learning and structural analysis have been used by some researchers to improve the analysis of WCE. Second, labeling video in WCE is less challenging than labeling individual frames, and some studies have focused on methods for deducing the diseases’ spatial and temporal distribution from video labels. Further, there should be a study of automated video trimming through video anomaly detection to minimize the time spent on reviewing.

Although deep learning techniques have shown high accuracy in WCE settings, many prior studies are limited by flaws in prior research data and low external validity. It will therefore be imperative to have further systematic and large-scale systematic studies in the future, which will help to make this technology more clinically applicable. In future studies, we will look into quality issues of WCE in order to determine areas that need improvement in order to better detect pathologies. Considering these observations, our goal is to design or obtain enhancement techniques and to test them in conjugation with physicians. Here we note that while traditional techniques like resolution enhancement have relatively small effect on deep learning performance, we will aim at contrast and texture enhancement to bring even further improvement to the deep learning models for WCE.

### Advancement of the State of the Art

In this paper, significant problems related to lesion detection, segmentation, and classification in WCE are proposed to extend the state of the art for deep learning applications. Unlike previous studies that concern the potential model accuracy, our work pays more attention to the interested clinical applications, including data-efficiency, real-time, and the spatial-temporal localization of the diseases. In the same breadth, we have also identified several research topics that don’t seem to have received much attention including semi-supervised learning and video anomaly detection which should be further explored for development in the future of WCE.

We also identify the potential for further interdisciplinary cooperation between AI practitioners and clinicians to enhance both the validity and applicability of deep learning platforms. In the course of this paper, we make a comparison of the techniques described and show how these innovations can fill the existing gaps in current studies to enhance the detection of GI diseases in WCE.

Lastly, by exploring some of the AI features like transfer learning and data-efficient methods, our future work will enhance deep learning methods in the subsequent work to support wider use of deep learning in actual clinics. Our proposal for the WCE survey is to extend the study on video enhancement for WCE using deep learning, and machine vision to enhance the detections. For more advancements on video enhancement and detection, readers can refer to our second paper, which provides detailed insights on these topics. ^32^

## Data Availability

We did not make use of any resources for datasets. The result under the outcome topic is based on the investigation conducted in the article. We examine and contrast the results based on the number of datasets and the CNN features used in the articles. We did not use any data sources in this survey and there was no need to use extra datasets.

## References

[1] Adewole S Fernandes P Jablonski J : *Graph Convolutional Neural Network For Weakly Supervised Abnormality Localization In Long Capsule Endoscopy Videos.* Piscataway: IEEE;2021; pp.388–399. Reference Source

[2] Väänänen A Haataja K Vehviläinen-Julkunen K : AI in healthcare: A narrative review [version 2; peer review: 1 not approved]. *F1000 Res.* 2021;10:6. 10.12688/f1000research.26997.2

[3] Goel N Kaur S Gunjan D : Investigating the significance of color space for abnormality detection in wireless capsule endoscopy images. *Biomed. Signal Process Control.* 2022;75:103624. 10.1016/j.bspc.2022.103624

[4] Cao J Yao J Zhang Z : *EFAG-CNN: Effectively Fused Attention Guided Convolutional Neural Network for WCE Image Classification.* IEEE;2021; pp.66–71. Reference Source

[5] Majtner T Brodersen JB Herp J : framework for autonomous detection and classification of Crohnʼs disease lesions in the small bowel and colon with capsule endoscopy. *Endosc. Int. Open.* 2021;09(09):E1361–E1370. 10.1055/a-1507-4980 PMC8367448 34466360

[6] Amiri Z Hassanpour H Beghdadi A : A Computer-Aided Method for Digestive System Abnormality Detection in WCE Images. *J. Healthc Eng.* 2021;2021:1–11. 10.1155/2021/7863113 34707798 PMC8545542

[7] Nam JH Oh DJ Lee S : Development and Verification of a Deep Learning Algorithm to Evaluate Small-Bowel Preparation Quality. *Diagnostics (Basel).* 2021;11(6):1127. 10.3390/diagnostics11061127 Reference Source 34203093 PMC8234509

[8] Stoleru CA Dulf EH Ciobanu L : Automated detection of celiac disease using Machine Learning Algorithms. *Sci. Rep.* 2022;12(1):4071. 10.1038/s41598-022-07199-z 35260574 PMC8904634

[9] Maissin A De Vallée R Flamant M : Multi-expert annotation of Crohn’s disease images of the small bowel for automatic detection using a convolutional recurrent attention neural network. *Endosc. Int. Open.* 2021;09(07):E1136. 10.1055/a-1468-3964 PMC8216776 34222640

[10] Mascarenhas M Ribeiro T Afonso J : Deep learning and colon capsule endoscopy: automatic detection of blood and colonic mucosal lesions using a convolutional neural network. *Endosc Int Open.* 2022;10(02):E171. 35186665 10.1055/a-1675-1941 PMC8850002

[11] Biradher S Aparna P : *Classification of Wireless Capsule Endoscopy Bleeding Images using Deep Neural Network.* Piscataway: IEEE;2021; pp.1–4. Reference Source

[12] Toonmana C Numpacharoen K Wiwatwattana N : *Bleeding Region Segmentation in Wireless Capsule Endoscopy Images by a Deep Learning Model: Initial Learning Rate and Epoch Optimization.* Piscataway: The Institute of Electrical and Electronics Engineers, Inc. (IEEE);2022. Reference Source

[13] Saraiva MM Ribeiro T Afonso J : Deep Learning and Device-Assisted Enteroscopy: Automatic Detection of Gastrointestinal Angioectasia. *Medicina (B Aires).* 2021;57(12). 10.3390/medicina57121378 PMC8706550 34946323

[14] Kora P Ooi CP Faust O : Transfer learning techniques for medical image analysis: A review. *Biocybern Biomed Eng.* 2022;42(1):79–107. 10.1016/j.bbe.2021.11.004 Reference Source

[15] Sunitha S Sujatha SS : *An Improved Bleeding Detection Method for Wireless Capsule Endoscopy (WCE) Images Based on AlexNet.* IEEE;2021; pp.11–15. Reference Source

[16] Muruganantham P Balakrishnan SM : Attention Aware Deep Learning Model for Wireless Capsule Endoscopy Lesion Classification and Localization. *J. Med. Biol. Eng.* 2022;42(2):157–168. 10.1007/s40846-022-00686-8

[17] Pascual G Laiz P García A : Time-based self-supervised learning for Wireless Capsule Endoscopy. *Comput. Biol. Med.* 2022;146:105631. 10.1016/j.compbiomed.2022.105631 35751203

[18] Saeed T Loo CK Kassim MSS : Ensembles of Deep Learning Framework for Stomach Abnormalities Classification. *Comput. Mater. Contin.* 2022;70(3):4357–4372. 10.32604/cmc.2022.019076

[19] Goel N Kaur S Gunjan D : Dilated CNN for abnormality detection in wireless capsule endoscopy images. *Soft Comput. (Berlin, Germany).* 2022;26(3):1231–1247. 10.1007/s00500-021-06546-y

[20] Ozturk S Ozkaya U : Residual LSTM layered CNN for classification of gastrointestinal tract. *J. Biomed. Inform.* 2021;113:103638. 10.1016/j.jbi.2020.103638 33271341

[21] Ozturk S Ozkaya U : Gastrointestinal tract classification using improved LSTM based CNN. *Multimed. Tools Appl.* 2020;79(39–40):28825–28840. 10.1007/s11042-020-09468-3

[22] Kim SH Hwang Y Oh DJ : Efficacy of a comprehensive binary classification model using a deep convolutional neural network for wireless capsule endoscopy. *Sci. Rep.* 2021;11(1):17479. 10.1038/s41598-021-96748-z Reference Source 34471156 PMC8410868

[23] Yogapriya J Chandran V Sumithra MG : Gastrointestinal Tract Disease Classification from Wireless Endoscopy Images Using Pretrained Deep Learning Model. *Comput. Math. Methods Med.* 2021;2021:1–12. 10.1155/2021/5940433 34545292 PMC8449743

[24] Zhao Q Yang W Liao Q : *AFA-RN: An Abnormal Feature Attention Relation Network for Multi-class Disease Classification in gastrointestinal endoscopic images.* IEEE;2021; pp.1–4. Reference Source

[25] Souaidi M Ansari M El : A New Automated Polyp Detection Network MP-FSSD in WCE and Colonoscopy Images Based Fusion Single Shot Multibox Detector and Transfer Learning. *IEEE access.* 2022;10:47124–47140. 10.1109/ACCESS.2022.3171238

[26] Saraiva MJM Afonso J Ribeiro T : Deep learning and capsule endoscopy: automatic identification and differentiation of small bowel lesions with distinct haemorrhagic potential using a convolutional neural network. *BMJ Open Gastroenterol.* 2021;8(1). 10.1136/bmjgast-2021-000753 PMC8477239 34580155

[27] Ribeiro T Saraiva MM Ferreira JPS : Artificial intelligence and capsule endoscopy: automatic detection of vascular lesions using a convolutional neural network. *Ann. Gastroenterol.* 2021;34(6):820–828. 10.20524/aog.2021.0653 34815648 PMC8596215

[28] Mohammed A Farup I Pedersen M : PS-DeVCEM: Pathology-sensitive deep learning model for video capsule endoscopy based on weakly labeled data. *Comput. Vis. Image Underst.* 2020;201:103062. 10.1016/j.cviu.2020.103062 Reference Source

[29] Saraiva MM Ferreira JPS Cardoso H : Artificial intelligence and colon capsule endoscopy: automatic detection of blood in colon capsule endoscopy using a convolutional neural network. *Endosc. Int. Open.* 2021;09(08):E1264–E1268. 10.1055/a-1490-8960 PMC8383083 34447874

[30] Gan T Yang Y Liu S : Automatic Detection of Small Intestinal Hookworms in Capsule Endoscopy Images Based on a Convolutional Neural Network. *Gastroenterol. Res. Pract.* 2021;2021:1–8. 10.1155/2021/5682288 34868306 PMC8635910

[31] Xu M : End-to-End Semi-Supervised Object Detection with Soft Teacher. Jun. 2021. Reference Source

[32] Habe TT Haataja K Toivanen P : Efficiency meets Accuracy: Benchmarking Object Detection Models for Pathology Detection in Wireless Capsule Endoscopy. *IEEE Access.* 2024; pp.1–1. 10.1109/ACCESS.2024.3456100

