# Supplementary material for: Review of Deep Learning Performance in Wireless Capsule Endoscopy Images for GI Disease Classification
Source: F1000Res. 2024 Sep 23;13:201. Originally published 2024 Mar 21. [Version 2] doi: 10.12688/f1000research.145950.2 (PMC11503939; doi:10.12688/f1000research.145950.2)
Supplement: Peer review history

# Reviewer report: Reviewer response for version 2

**DOI:** 10.5256/f1000research.172097.r326137

**Published:** 2024-10-23

**Authors:** Gulshan Kumar

**Affiliations:** 1 Shaheed Bhagat Singh State University, Firozpur, India

Authors addressed my concerns. Recommended for indexing.

Is the review written in accessible language?

Yes

Are all factual statements correct and adequately supported by citations?

Yes

Are the conclusions drawn appropriate in the context of the current research literature?

Partly

Is the topic of the review discussed comprehensively in the context of the current literature?

Yes

Reviewer Expertise:

AI techniques and its applications area, Intrusion Detection, Network Security

I confirm that I have read this submission and believe that I have an appropriate level of expertise to confirm that it is of an acceptable scientific standard.

# Reviewer report: Reviewer response for version 2

**DOI:** 10.5256/f1000research.172097.r326138

**Published:** 2024-10-16

**Authors:** Reena Gunjan, Prof. Dr. Vrushali Rampure

**Affiliations:** 1 Computer Science & Engineering Department, MIT Art, Design and Technology University, Pune, Pune, MAHARASHTRA, India; 2 Electronics Department, Maharashtra Academy of Naval Education & Training, Pune, Maharashtra, India

The new version is reviewed and the suggestions have been implemented. The revisions are appropriate. It is approved. Please proceed.

Is the review written in accessible language?

Yes

Are all factual statements correct and adequately supported by citations?

Yes

Are the conclusions drawn appropriate in the context of the current research literature?

Partly

Is the topic of the review discussed comprehensively in the context of the current literature?

Partly

Reviewer Expertise:

Deep Learning, Medical Imaging

We confirm that we have read this submission and believe that we have an appropriate level of expertise to confirm that it is of an acceptable scientific standard.

# Reviewer report: Reviewer response for version 1

**DOI:** 10.5256/f1000research.159975.r280170

**Published:** 2024-09-16

**Authors:** Reena Gunjan

**Affiliations:** 1 Computer Science & Engineering Department, MIT Art, Design and Technology University, Pune, Pune, MAHARASHTRA, India

**Brief Description and Relevance**:

 This paper presents a comprehensive review of the current trends and future directions in applying deep learning techniques to wireless capsule endoscopy (WCE).  The paper addresses challenges such as low-resolution images, artifacts, and annotation difficulties while discussing deep learning methodologies such as transfer learning, attention mechanisms, and semi-supervised learning. Given the increasing use of AI in medical diagnostics, this review is highly relevant for researchers, clinicians, and engineers aiming to leverage AI advancements in GI tract diagnostics.

**Major Points:**

**1) Clarifying Challenges and Limitations**: The paper does a good job of highlighting some of the challenges faced by deep learning in WCE, such as data annotation and artifacts. However, it could be more explicit about how current methods fall short in specific areas. For example, while deep learning has demonstrated success in segmentation and lesion detection, what are the common pitfalls in classification tasks.

**Recommendation**: Provide concrete examples of how deep learning models currently underperform in specific GI tract disease classifications and what factors contribute to these limitations.

**2) Future Directions**: The section on future directions offers an optimistic outlook but could benefit from more concrete and actionable steps. For instance, while data-efficient learning is a promising direction, how can researchers practically implement semi-supervised algorithms in WCE datasets, given the unique challenges of this field? 

**Recommendation**: Include more specific methodologies or frameworks that could be adopted for data-efficient learning and video anomaly detection in future research.

**3) Encouraging Collaborative Efforts**: The paper briefly touches upon the role of researchers, clinicians, and engineers in advancing WCE technology, but it could further emphasize the need for interdisciplinary collaboration. Engaging these diverse fields will be crucial for ensuring that deep learning models are both technically sound and clinically relevant.

**Recommendation**: Highlight the importance of collaboration between AI researchers, gastroenterologists, and medical imaging experts and suggest ways in which such partnerships could be fostered.

**Minor Points:**

1. Some of the terminology used, particularly in relation to deep learning concepts (e.g., "structural understanding of data," "spatiotemporal location of diseases"), may be unclear to readers unfamiliar with the field. Simplifying or briefly explaining these terms would improve the paper's accessibility.
**Recommendation**: Include a brief glossary or explanations of key terms, particularly those related to deep learning and AI, to make the article more accessible to a wider audience.
2. **Improvement Suggestions for Image Quality Section**: The authors mention that resolution enhancement may not be necessary but suggest focusing on contrast and texture enhancement. This is a valid point, but more detail could be provided about the specific challenges that low contrast or poor texture causes in deep learning models for WCE.
**Recommendation**: Expand the discussion on how contrast and texture improvements can directly benefit the performance of deep learning models, potentially through quantitative examples or referencing specific studies that have addressed these image quality issues

**Overall Evaluation:**

 The article provides an insightful review of the current state and future potential of deep learning in wireless capsule endoscopy.  With revisions to clarify complex concepts, integrate technical comparisons earlier, and provide more actionable future directions, this paper can serve as a valuable resource for advancing research and clinical applications in WCE.

Is the review written in accessible language?

Yes

Are all factual statements correct and adequately supported by citations?

Yes

Are the conclusions drawn appropriate in the context of the current research literature?

Partly

Is the topic of the review discussed comprehensively in the context of the current literature?

Partly

Reviewer Expertise:

Deep Learning, Medical Imaging

I confirm that I have read this submission and believe that I have an appropriate level of expertise to confirm that it is of an acceptable scientific standard, however I have significant reservations, as outlined above.

# Response

**Published:** 2024-09-17

**Authors:** Tsedeke Temesgen Habe

**Affiliations:** Computer Science, University of Eastern Finland, Helsinki, Finland

Thank you for your insightful comments. We have carefully addressed all the points raised, and our responses are as follows:

1. **Clarifying Challenges and Limitations**: We have revised the section on challenges and limitations to provide specific examples of how current deep learning models underperform in GI disease classification. The updated section now includes a discussion on misclassification between similar-looking lesions and the role of dataset imbalance, which contributes to these limitations. This expansion provides more concrete insights into the pitfalls of current methods.
2. **Future Directions**: The future directions section has been expanded to include more actionable steps for researchers. We have added specific methodologies and frameworks for implementing semi-supervised learning and video anomaly detection, particularly in the context of WCE datasets. These revisions offer clearer guidance on how future research can tackle the unique challenges of WCE.
3. **Encouraging Collaborative Efforts**: We have added a new section that emphasizes the importance of interdisciplinary collaboration between AI researchers, clinicians, and engineers. This section discusses the necessity of fostering partnerships and provides practical suggestions for encouraging such collaboration, including shared datasets and interdisciplinary projects.
4. **Image Quality Section**: We have expanded the discussion on how contrast and texture improvements can benefit the performance of deep learning models, referencing specific studies that address these image quality challenges. This revision adds depth to the image quality section, illustrating how preprocessing techniques can directly enhance model performance.

# Reviewer report: Reviewer response for version 1

**DOI:** 10.5256/f1000research.159975.r306936

**Published:** 2024-09-16

**Authors:** Gulshan Kumar

**Affiliations:** 1 Shaheed Bhagat Singh State University, Firozpur, India

The paper provides a comprehensive review of the current trends and future directions in deep learning for wireless capsule endoscopy (WCE). While it covers important advancements such as transfer learning, attention mechanisms, multimodal learning, automated lesion detection, interpretability and explainability, data augmentation, and edge computing,. The discussion on challenges and limitations is insightful, yet the paper would benefit significantly from a more explicit statement of how it advances the state of the art, addresses existing gaps,  Additionally, more detailed comparisons of the methodologies and their practical implications for clinical practice would enhance the paper's relevance and impact. Therefore, a major revision is necessary to clearly define the paper's contribution and strengthen its analysis and conclusions.

Is the review written in accessible language?

Yes

Are all factual statements correct and adequately supported by citations?

Yes

Are the conclusions drawn appropriate in the context of the current research literature?

Partly

Is the topic of the review discussed comprehensively in the context of the current literature?

Yes

Reviewer Expertise:

AI techniques and its applications area, Intrusion Detection, Network Security

I confirm that I have read this submission and believe that I have an appropriate level of expertise to confirm that it is of an acceptable scientific standard, however I have significant reservations, as outlined above.

# Response

**Published:** 2024-09-17

**Authors:** Tsedeke Temesgen Habe

**Affiliations:** Computer Science, University of Eastern Finland, Helsinki, Finland

Thank you for your valuable feedback, which has greatly helped us enhance the clarity and impact of our paper. Below is our response to the major points raised:

1. **Advancing the State of the Art**: We have updated the conclusion section to explicitly highlight how our paper advances the state of the art in applying deep learning techniques to Wireless Capsule Endoscopy (WCE). We have added a detailed discussion on how our review addresses existing gaps in the literature, particularly in lesion detection, segmentation, and classification, which are underexplored areas in current research.
2. **Comparison of Methodologies**: In response to your suggestion, we expanded the Result section comparing deep learning methodologies. The updated section now includes a more detailed analysis of the practical implications for clinical practice, considering factors such as computational efficiency, model interpretability, and how each method could impact real-world clinical workflows.
3. **Strengthening Analysis and Conclusions**: We have reworked the conclusions to better align with the current research literature, providing a clearer summary of how the methods reviewed in the paper can directly contribute to advancing WCE technology. This revision also includes more concrete recommendations for both researchers and clinicians.

We appreciate your constructive feedback and believe these changes have strengthened the overall quality and relevance of the paper.
